# Dengue Cases Presenting to the Emergency Department of a Tertiary Care Hospital in Late 2021: A Cross-Sectional Study in Karachi

**DOI:** 10.3389/ijph.2024.1606753

**Published:** 2024-02-15

**Authors:** Saima Mushtaq, Muhammad Tarish Abro, Hassan ul Hussain

**Affiliations:** ^1^ Jinnah Post Graduate Medical Centre, Karachi, Pakistan; ^2^ Sindh Medical College, Jinnah Sindh Medical University, Karachi, Pakistan; ^3^ Dow Medical College, Dow University of Health Sciences, Karachi, Pakistan

**Keywords:** dengue fever, COVID-19, emergency department, tropical diseases, Asia

## Abstract

**Objectives:** Dengue Fever (DF) is an arboviral disease caused by the Dengue virus (DENV). This study aims to assess the association of dengue prevalence with patients’ residential areas and hematological laboratory findings (Total platelet count, immunoglobulins, and dengue antigens) during COVID-19 pandemic.

**Methods:** A retrospective study was conducted at the Emergency department of Jinnah Postgraduate Medical Center, Karachi from October to December 2021. All the patients irrespective of their ages presenting to the ED with either of the following complaints: fever; GIT problems; vomiting; body ache; bleeding were included in our study.

**Results:** Our study comprised 189 patients in total in which the females (*n* = 172) outnumbered the males (*n* = 17). Out of all, 84.7% of the patients were febrile having a low-grade fever on average. Korangi district had the most dengue cases, while Keamari had the fewest. There was no significant association reported between mean platelet count, mean TLC, and mean hemoglobin levels with dengue positivity.

**Conclusion:** There is a concerning rise in DF cases in Karachi, particularly in the Korangi district. Despite the COVID-19 pandemic, DF demands urgent attention.

## Introduction

Dengue Fever (DF) is an arboviral disease caused by the Dengue virus (DENV), and it is a serious public health problem owing to its rapid spread. The most prevalent mosquito vectors for the transmission of the infection are *Aedes aegypti* and *Aedes albopictus* [1]. According to a report, DF is estimated to affect 3.9 billion people worldwide and out of which 70% burden exists in Asia [2]. Countries that are worst affected by the DENV are Pakistan, Bangladesh, India, and Sri Lanka [3]. Pakistan is endemic to the DENV for the last 30 years [4]. The first incidence of DF in Pakistan was recorded in Karachi in 1994 [5]. Between 2015 and 2019, there were 90,400 dengue cases and 169 fatalities in Pakistan [4]. Similarly, 3,442 confirmed dengue cases had been reported in 2020 [6]. According to the news, a substantial increase in dengue infections was recorded in the latter 3 months of 2021, with 48,906 cases reported as of November 25, 2021, with 183 fatalities [7, 8].

DF is a single disease that can manifest in a variety of ways, from asymptomatic seroconversion to moderate or severe DF, which can result in shock, severe bleeding, and even death [9, 10]. Dengue hemorrhagic fever (DHF) and Dengue shock syndrome (DSS) are the two most common clinical complications caused by DF. DHF and DSS are reversible vascular complications of DF that cause severe thrombocytopenia and increased vascular permeability [11]. For this reason, a decrease in platelet and white blood cell counts, as well as an increase in plasma leakage, are regarded as highly efficient indicators of dengue infection [12]. Dengue infection symptoms include vomiting, body aches, abdominal pain, and bleeding, in addition to a high temperature [13].

Pakistan is already dealing with the COVID-19 pandemic and another dengue outbreak would be disastrous for the country’s already debilitated healthcare infrastructure. Moreover, since DF and COVID-19 have similar clinical symptoms, they can readily exist as co-infection [14]. For this reason, it is important to have fore-hand preparation of the healthcare system as well as the authorities to avoid further harm and damage.

This study aims to assess the profile of dengue patients presenting to the Emergency Department (ED) of a tertiary care hospital during COVID-19 pandemic. It includes determining their sociodemographic factors including the patients’ residential area to determine dengue endemic regions of Karachi, and the hematological laboratory findings including total platelet count, immunoglobulin M (IgM), immunoglobulin G (IgG), and non-structural protein 1 (NS1) antigens with the associated symptoms of DF.

## Methods

### Design and Setting

A retrospective study was conducted at the ED of Jinnah Postgraduate Medical Center (JPMC) from October to December 2021. JPMC is a tertiary care public hospital located in Karachi, Pakistan. The ethical approval of the study was obtained from the hospital’s Institutional Review Board (IRB) (NO.F.281/2022-GENL/242/JPMC).

### Inclusion Criteria

All the patients irrespective of their ages presenting to the ED with either of the following complaints: fever; GIT problems; vomiting; body ache; bleeding were included in our study. Patients who did not give consent or had chronic diseases or gave incomplete information were excluded from our study. We only included the patients who met the inclusion criteria.

### Data Collection

The data were collected from the ED of JPMC. It included patients’ demographical details (age, gender, and residential area), presenting complaints, lab tests, temperature, and dengue test reports. Dengue infection were confirmed through standard Dengue NS1 antigen enzyme-linked immunosorbent assay (ELISA) and standard Dengue IgM or IgG Capture ELISA kits (Standard Diagnostics Inc, Kyonggi-do Korea). Patient’s identities, personal information, and reports were kept confidential and only the primary investigators were given access to that data. All the ethical considerations were kept in mind throughout the study.

### Statistical Analysis

IBM Corp. Released 2016. IBM SPSS Statistics for Windows, Version 24.0. Armonk, NY: IBM Corp. was used to perform the data analysis. The analysis was performed in four stages. First, the frequencies of all the variables were determined. Then, mean ± standard deviation (SD) values of age, temperature, hemoglobin levels, total leukocyte count (TLC), and platelet count were calculated using descriptive statistics. To evaluate the association between categorical variables, we performed Pearson’s chi-squared tests. Lastly, we performed the Independent Samples T-test to determine the relationship between categorical and continuous variables. A *p*-value of <0.05 was considered significant, statistically.

## Results

### Demographical and Baseline Characteristics of the Patients

**FIGURE 1:**
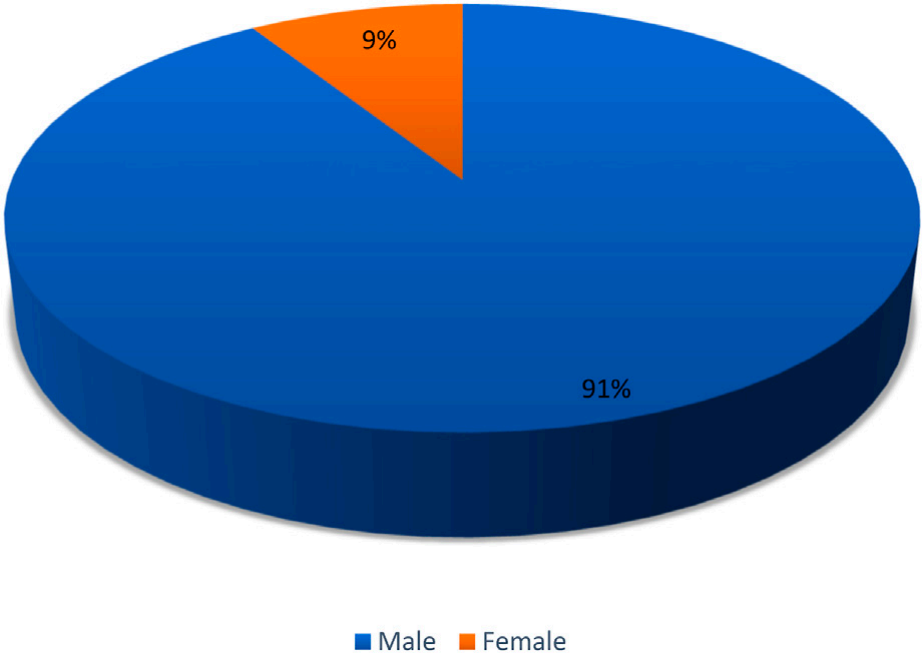
Gender-based distribution of the patients (Karachi, Pakistan. 2021).

**FIGURE 2:**
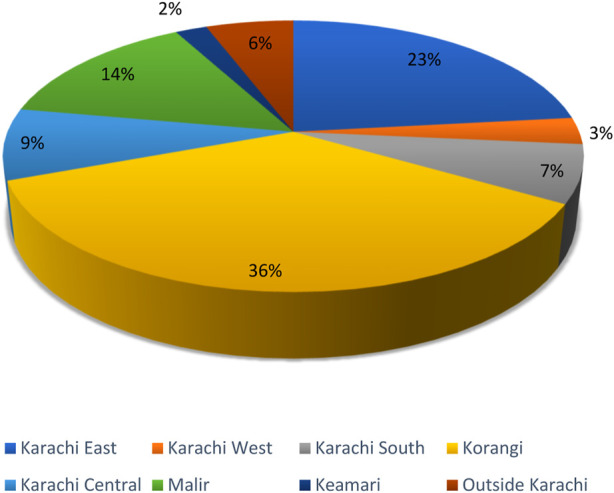
District-wise distribution of the patients (Karachi, Pakistan. 2021).

Our study comprised 189 patients in total in which the females (*n* = 172) outnumbered the males (*n* = 17) as depicted by the pie chart in Figure 1. The mean age of the participants was reported to be 31.59 ± 14.68 years. The participants were reported to have a low fever on average. Average platelet count and other hematological parameters are mentioned in Table 1. Upon district-wise distribution of the participants, more than one-third of them (36%) were the residents of Korangi district. The second and third most common districts were Karachi East (23%) and Malir (14%), respectively. They were followed by Karachi Central, Karachi South, areas outside Karachi, Karachi West, and Keamari (Figure 2).

**TABLE 1:** Means of ages, body temperature, and hematological parameters of patients (Karachi, Pakistan. 2021).

Participants’ characteristics	Mean ± SD
Age (years)	31.59 ± 14.68
Temperature (°F)	100.59 ± 1.61
Platelet count (mcL)	108217.99 ± 152660.41
Hemoglobin level (g/dL)	13.20 ± 2.51
Total leukocyte count (cell/ccm)	5.18 ± 2.87

### Dengue Diagnosis

The diagnosis of the DENV was made on the basis of different antigens’ serum positivity and negativity. Out of 159 patients who were positive for DENV, around three-quarters of them (73.5%) were positive for NS1 antigen, while a few (2.6%, 5.3%) were positive for IgM and IgG respectively. The remaining ones (2.6%) were positive for both IgM and IgG (Table 2).

**TABLE 2:** Antigen-wise division of dengue diagnosis (Karachi, Pakistan. 2021).

Antigen	Subgroup	No. of participants (%)
NS1	Positive	139 (73.5)
	Negative	28 (14.8)
IgM	Positive	5 (2.6)
	Negative	—
IgG	Positive	10 (5.3)
	Negative	1 (0.5)
IgG/IgM	Positive	5 (2.6)
	Negative	1 (0.5)

### Signs and Symptoms

Upon arrival at the hospital, the participants were checked for their dengue status. A substantial number of subjects (84.1%) tested positive for the DENV. Out of all, 84.7% of the patients were febrile having a low-grade fever on average. A huge majority (>90%) of the patients did not have the following signs and symptoms: headache; vertigo; rash; rigors; chills; fits; gallbladder wall (GBW) thickening; nausea; altered level of consciousness (ALOC); hematuria; per rectal bleeding; oral bleeding; epistaxis; generalized body weakness; diarrhea; shortness of breath (SOB); cough; epigastric pain; hematemesis. Nonetheless, generalized body ache (31.7%), abdominal pain (14.8%), and vomiting (29.6%) were more prevalent amongst the patients in comparison with other symptoms (Table 3).

**TABLE 3:** Signs and symptoms in patients (Karachi, Pakistan. 2021).

Signs/symptoms	Subgroup	No. of participants (%)
Dengue status	Positive	159 (84.1)
	Negative	30 (15.9)
Fever status	Febrile	160 (84.7)
	Afebrile	29 (15.3)
Headache	Yes	6 (3.2)
	No	183 (96.8)
Vertigo	Yes	2 (1.1)
	No	187 (98.9)
Rash	Yes	4 (2.1)
	No	185 (97.9)
Rigors	Yes	2 (1.1)
	No	187 (98.9)
Chills	Yes	10 (5.3)
	No	179 (94.7)
Fits	Yes	2 (1.1)
	No	187 (98.9)
Gall bladder wall thickening	Yes	6 (3.2)
	No	183 (96.8)
Nausea	Yes	13 (6.9)
	No	176 (93.1)
Altered level of consciousness	Yes	3 (1.6)
	No	186 (98.4)
Hematuria	Yes	2 (1.1)
	No	187 (98.9)
Per rectal bleeding	Yes	3 (1.6)
	No	186 (98.4)
Oral bleeding	Yes	7 (3.7)
	No	182 (96.3)
Epistaxis	Yes	13 (6.9)
	No	176 (93.1)
Generalized body weakness	Yes	8 (4.2)
	No	181 (95.8)
Diarrhea	Yes	14 (7.4)
	No	175 (92.6)
Shortness of breath	Yes	5 (2.6)
	No	184 (97.4)
Cough	Yes	3 (1.6)
	No	186 (98.4)
Generalized body ache	Yes	60 (31.7)
	No	129 (68.3)
Abdominal pain	Yes	28 (14.8)
	No	161 (85.2)
Epigastric pain	Yes	6 (3.2)
	No	183 (96.8)
Vomiting	Yes	56 (29.6)
	No	133 (70.4)
Hematemesis	Yes	7 (3.7)
	No	182 (96.3)

### Association of Dengue With Categorical and Continuous Variables

Our study reported a significant association between the patients’ district of residence and dengue status with Korangi having the greatest number of dengue cases (*n* = 62) and Keamari having the least ones (*n* = 3) (*p* = 0.053). According to our study, symptoms of vertigo and vomiting were significantly lower in dengue positive patients (*p* = 0.024; *p* = 0.010). Nevertheless, our study reported no significant association between dengue positivity and the following signs and symptoms: headache; rash; rigors; chills; fits; GBW thickening; nausea; ALOC; hematuria; per rectal bleeding; oral bleeding; epistaxis; generalized body weakness; diarrhea; SOB; cough; generalized body ache; abdominal pain; epigastric pain; hematemesis. Moreover, there was no significant gender difference reported (Table 4). There was no significant association reported between mean platelet count, mean TLC, and mean hemoglobin levels with dengue positivity (Table 5).

**TABLE 4:** Association of dengue positivity with gender, district, and signs/symptoms of patients (Karachi, Pakistan. 2021).

Variable	Subgroup	No. of participants investigated	No. of participants positive for DF	*p*-value
Gender	Male	61	55	0.117
	Female	128	104	
District	Karachi East	44	35	0.053
	Karachi West	6	6	
	Karachi South	13	8	
	Karachi Central	16	11	
	Korangi	68	62	
	Malir	27	25	
	Kemari	4	3	
	Outside Karachi	11	9	
Fever status	Febrile	160	134	1.000
	Afebrile	29	25	
Headache	Yes	6	6	0.592
	No	183	153	
Vertigo	Yes	2	0	0.024
	No	187	159	
Rash	Yes	4	2	0.119
	No	185	157	
Rigors	Yes	2	2	1.000
	No	187	157	
Chills	Yes	10	8	0.661
	No	179	151	
Fits	Yes	2	1	0.293
	No	187	158	
Gall bladder wall thickening	Yes	6	4	0.243
	No	183	155	
Nausea	Yes	13	13	0.228
	No	176	146	
Altered level of consciousness	Yes	3	2	0.406
	No	186	157	
Hematuria	Yes	2	2	1.000
	No	187	157	
Per rectal bleeding	Yes	3	3	1.000
	No	186	156	
Oral bleeding	Yes	7	6	1.000
	No	182	153	
Epistaxis	Yes	13	12	0.696
	No	176	147	
Generalized body weakness	Yes	8	7	1.000
	No	181	152	
Diarrhea	Yes	14	10	0.244
	No	175	149	
Shortness of breath	Yes	5	3	0.179
	No	184	156	
Cough	Yes	3	3	1.000
	No	186	156	
Generalized body ache	Yes	60	50	0.839
	No	129	109	
Abdominal pain	Yes	28	24	1.000
	No	161	135	
Epigastric pain	Yes	6	5	1.000
	No	183	154	
Vomiting	Yes	56	53	0.010
	No	133	106	
Hematemesis	Yes	7	7	0.599
	No	182	152	

**TABLE 5:** Association of dengue positivity with hematological parameters of patients (Karachi, Pakistan. 2021).

Hematological marker	Dengue		Mean difference	*p*-value
	Positive	Negative		
Mean platelet count ± SD	107862.89 ± 149369.32	110100.00 ± 171814.12	2237.11	0.942
Mean total leukocyte count ± SD	5.04 ± 2.84	6.44 ± 3.15	1.39	0.307
Mean hemoglobin level ± SD	13.13 ± 2.49	16.00 ± 0.00	2.87	0.264

## Discussion

In this retrospective study, the profile of dengue patients has been assessed whilst COVID-19 was still in effect. In low- and middle-income nations like Pakistan, the co-occurrence of these two infectious diseases might have a significant adverse effect on both the population and the economy [15].

Dengue infections were confirmed through antigen and antibody detection. Several patients tested positive for NS1 antigen but relatively few tested positive for IgG and IgM antibodies. This result shows that the NS1 antigen test was a more potent diagnostic technique for the identification of DF as compared to dengue IgM and IgG antibodies detection.

In contrast to other common symptoms of DF, individuals were more likely to have generalized body ache, abdominal pain, and vomiting. This result was in accordance with a study conducted in Karachi [16].

Additionally, the area of residence of each participant was also included, and it was separated into the districts of Karachi. This was done to determine where the majority of cases originated, which will assist in identifying dengue endemic locations. The results indicated that most of the cases originated in the Korangi district, followed by Karachi East and Malir district. With a population density of 3,900 people per square kilometer (10,000/sq mi), Karachi is an extremely populated city [17]. The two primary causes of the rise of dengue virus infection in tropical emerging nations are an unpredicted rise in population and unplanned urban expansion that leads to major public health problems [18].

This study of patients with dengue showed that the issue of DF has grown even more serious as authorities are busy dealing with COVID-19. Our study concluded that Korangi district was determined to be the most significant dengue-endemic area in Karachi. It is important that the general population, and especially healthcare professionals, should be aware of preventive techniques since doing so will help in the efficient diagnosis that allows appropriate patient care, generation of accurate epidemiological data, and implementation of effective public health actions.

This study shows that there were more female targets than male targets among the affected patients. This is in contrast to many researches where the majority of the patients were male [19, 20]. Social, cultural, and environmental factors may have led to the phenomena of gender specificity in relation to dengue infection. This could be due to society’s neglect towards women’s health problems along with amenia and their debilitating immune system [21].

The district-wise distribution of dengue patients in this study makes it one of its kind to identify the dengue endemic regions of the city. Moreover, this study provides a better picture of dengue cases whilst COVID-19 was at its peak. The limitations of this study include a small sample size and a single-centered design. Furthermore, the patients were not followed up to assess the long-term effects of dengue infection.

### Conclusion

This study reveals a worrying surge in DF cases in Karachi, especially concentrated in the Korangi district. Out of 159 DENV-positive patients, 73.5% tested positive for NS1 antigen, while 2.6% and 5.3% tested positive for IgM and IgG, respectively. Despite the COVID-19 pandemic, DF demands urgent attention. To effectively combat this growing threat, health authorities must prioritize DF control alongside COVID-19 management. Targeted interventions focused on Korangi and areas with high female populations are crucial. Implementing robust public health actions like mosquito control programs are essential. Furthermore, studies with larger sample sizes and long-term patient follow-up are needed. By taking these decisive steps, we can safeguard the health of Karachi’s residents and effectively manage the rising tide of DF.
